# LOXL3 Silencing Affected Cell Adhesion and Invasion in U87MG Glioma Cells

**DOI:** 10.3390/ijms22158072

**Published:** 2021-07-28

**Authors:** Talita de S. Laurentino, Roseli da S. Soares, Antonio M. Lerario, Suely K. N. Marie, Sueli M. Oba-Shinjo

**Affiliations:** 1 Cellular and Molecular Biology Laboratory (LIM 15), Neurology Department, Faculdade de Medicina (FMUSP), Universidade de Sao Paulo, Sao Paulo 01246-000, SP, Brazil; roselicem@yahoo.com.br (R.d.S.S.); sknmarie@usp.br (S.K.N.M.); 2 Department of Internal Medicine, Division of Metabolism, Endocrinology and Diabetes, University of Michigan, Ann Arbor, MI 48109, USA; amlerario@gmail.com

**Keywords:** LOXL3, lysyl oxidase, glioblastoma, cytoskeleton, extracellular matrix, cell death, endocytosis

## Abstract

Lysyl oxidase-like 3 (LOXL3), belonging to the lysyl oxidase family, is responsible for the crosslinking in collagen or elastin. The cellular localization of LOXL3 is in the extracellular space by reason of its canonical function. In tumors, the presence of LOXL3 has been associated with genomic stability, cell proliferation, and metastasis. In silico analysis has shown that glioblastoma was among tumors with the highest *LOXL3* expression levels. *LOXL3* silencing of U87MG cells by siRNA led to the spreading of the tumor cell surface, and the transcriptome analysis of these cells revealed an upregulation of genes coding for extracellular matrix, cell adhesion, and cytoskeleton components, convergent to an increase in cell adhesion and a decrease in cell invasion observed in functional assays. Significant correlations of *LOXL3* expression with genes coding for tubulins were observed in the mesenchymal subtype in the TCGA RNA-seq dataset of glioblastoma (GBM). Conversely, genes involved in endocytosis and lysosome formation, along with MAPK-binding proteins related to focal adhesion turnover, were downregulated, which may corroborate the observed decrease in cell viability and increase in the rate of cell death. Invasiveness is a major determinant of the recurrence and poor outcome of GBM patients, and downregulation of LOXL3 may contribute to halting the tumor cell invasion.

## 1. Introduction

Glioblastoma (GBM), the most aggressive and common type of malignant brain tumor [1], is characterized by rapid growth and invasion, neovascularization, and necrosis [2]. Initially, gliomas were classified based only on their histologic characteristics. However, in 2016, the World Health Organization restructured the classification of central nervous system tumors, dividing astrocytomas into two groups: diffuse astrocytic tumors (including GBM) and other astrocytic tumors. In addition, molecular features, including isocitrate dehydrogenase (NADP(+)) 1/2 (IDH1/2) mutations, have been incorporated into the classification of gliomas [3]. In addition to the World Health Organization classification, The Cancer Genome Atlas (TCGA) network presented the genetic signatures of GBM. Based on molecular alterations, GBMs were subdivided into classical, mesenchymal, and proneural molecular subtypes [4,5]. Maximal tumor resection, followed by radiotherapy and chemotherapy with temozolomide (TMZ), is currently the standard treatment for patients with GBM. Nonetheless, a high recurrence rate and resistance to TMZ frequently occur in patients with GBM, resulting in a median overall survival of 15 months [6].

Lysyl oxidase-like 3 (LOXL3), a copper-dependent amine oxidase, belongs to the lysyl oxidase family which comprises four other members (LOX, LOXL1, LOXL2, and LOXL4). These proteins are responsible for the oxidative deamination of the amine group of lysine residues in tropocollagen, converting collagen or elastin monomers into insoluble fibers [7]. The lysyl oxidase family is divided into two subgroups according to the similarities in the N-terminal region. LOX and LOXL1 present pro-sequences in the N-terminal, thereby leading to their secretion as inactive pro-enzymes. In contrast, LOXL2, LOXL3, and LOXL4 contain four scavenger receptor cysteine-rich domains [8]. The C-terminal domain presents catalytic activity and is conserved across the lysyl oxidase family proteins. This region contains a copper-binding motif, lysyl-tyrosyl-quinone cofactor residues, and a cytokine receptor-like domain. Conversely, the N-terminal region varies among the members of the lysyl oxidase family [9,10].

Owing to its amine oxidase activity, LOXL3 is localized in the extracellular space. Based on its predicted structure, LOXL3 can be secreted in the extracellular matrix (ECM) and is processed by bone morphogenetic protein 1 (BMP1) [11]. Conversely, LOXL3 can also be translocated to the nucleus due to a bipartite nuclear localization signal (residues 293–311), suggesting additional roles [11,12,13]. Other functions beyond amine oxidase activity have been attributed to LOXL3, such as oxidation of fibronectin and consequently activation of the integrin pathway [14]. Moreover, it may act as a dual enzyme with deacetylation and deacetylimination activities of STAT3 to control the inflammatory response [15]. In tumors, LOXL3 interacts with SNAIL, a transcription factor involved in the epithelial–mesenchymal transition process, thereby contributing to metastasis and tumor progression [16]. Furthermore, LOXL3 maintains genomic stability in melanoma by association with oncogenic BRAF in melanogenesis and promotes sustained proliferation [17]. It is also upregulated in various tumors, such as gastric cancer cells, breast cancer, myeloproliferative neoplasms, ovarian carcinoma, and colorectal cancer [13,18,19,20,21,22], suggesting that it may be a target candidate for the treatment of tumors.

Although LOXL3 plays different roles in tumorigenesis and in tumor progression, there are no studies investigating the expression of LOXL3 expression in GBM. Therefore, we analyzed the contribution of LOXL3 to the pathogenesis and aggressiveness of GBM through in silico analysis and cellular assays using the U87MG glioma cell line as a model system.

## 2. Results

### 2.1. LOXL3 Is Overexpressed in Human GBM

**Figure 1:**
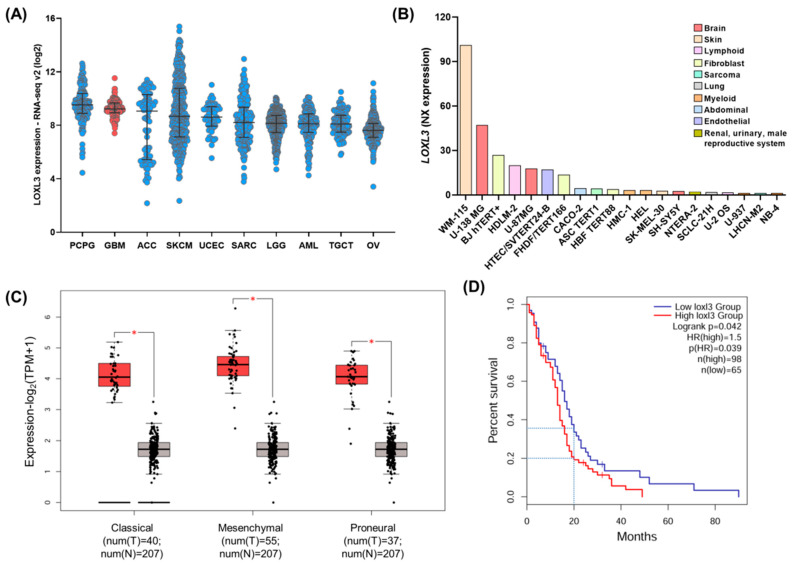
*LOXL3* expression in cancer tissues and cell lines. (**A**) *LOXL3* expression levels in 10 different types of cancer using data obtained from of The Cancer Genome Atlas (TCGA) database of Pan-cancer Atlas. The graph represents the log2 scale of the RPKM of tumors with higher median values. Bars indicate the median and interquartile ranges of each group. (**B**) *LOXL3* expression in different cell lines using data obtained from The Human Protein Atlas. The results are reported as normalized expression (NX) values ≥ 1 of immortalized cancer cells. (**C**) Boxplots illustrating *LOXL3* expression in GBM molecular subtypes and normal brain tissues in TCGA and GTEx, respectively, based on GEPIA2 analysis. The *Y*-axis represents the log2 (TPM+1) of *LOXL3* expression levels. Statistical differences between GBM (T) and normal brain (N) are represented by asterisk: * *p* < 0.05 denotes statistically significant differences. (**D**) Overall survival rate by Kaplan–Meier analysis in GBM cases separated according to *LOXL3* expression (40% with higher and lower levels). Analysis was performed using GEPIA2. Abbreviations: ACC, adrenocortical carcinoma; AML, acute myeloid leukemia; GBM, glioblastoma; GEPIA2, Gene Expression Profiling Interactive Analysis 2; GTEx, Genotype-Tissue Expression; LGG, low-grade gliomas; *LOXL3*, lysyl oxidase-like 3; OV, serous ovarian cancer; PCPG, pheochromocytoma; RPKM, reads per kilobase per million; SARC, sarcoma; SKCM, cutaneous melanoma; TGCT, testicular germ cell tumor; TPM, transcripts per million.

Initially, we investigated the expression of LOXL3 in 32 different types of cancer using 10,967 samples from the TCGA RNA-seq database. High LOXL3 expression levels were detected in 10 types of cancer, including GBM (Figure 1A, in red). The cell lines derived from gliomas (U138MG and U87MG) were in the top five cell lines presenting the highest LOXL3 expression levels among the 64 human cell lines of the HPA study (Figure 1B). The GEPIA2 web tool was used to compare the levels of LOXL3 in normal brain samples and GBM cases according to the molecular mesenchymal, classical, and proneural subtypes (Figure 1C). All three GBM subtypes presented a significantly higher expression of LOXL3 compared with the normal brain. Although the differences detected among the GBM subtypes were not statistically significant, the mesenchymal subtype presented the highest LOXL3 expression levels compared with the classical and proneural subtypes. Moreover, the levels of LOXL3 expression affected the outcome of patients with GBM. Patients who presented an upregulation LOXL3 by ≤40% of its highest expression level were associated with reduced overall survival versus those who exhibited downregulation of the same range (*p* = 0.039, Kaplan–Meier survival analysis) (Figure 1D). Additionally, the impact of the expression levels of LOXL3 on survival at 20 months was clear, as only 20% and 35% of patients survived in the high- and low-expression groups, respectively. Collectively, these results suggest that LOXL3 is overexpressed in GBM, impacting overall survival and playing a still unexplored role in this tumor type.

### 2.2. LOXL3 Silencing by siRNA

**Figure 2:**
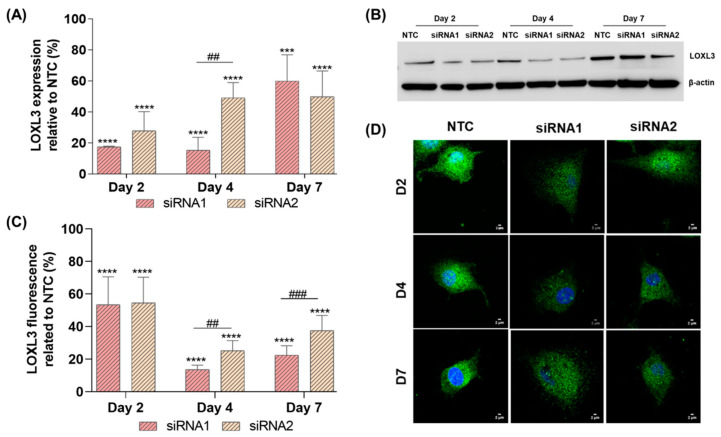
Downregulation of *LOXL3* expression by siRNA in U87MG cells. (**A**) RT-qPCR analysis of *LOXL3* expression relative to control (NTC) at 2, 4, and 7 days after transfection with two different siRNA sequences (siRNA1 and siRNA2). (**B**) Western blotting analysis of LOXL3 expression in the control (NTC) group and after silencing with siRNA1 and siRNA2. β-actin was used as loading control. LOXL3: 83 kDa; β-actin: 42 kDa. (**C**) Quantification of LOXL3 fluorescence in control (NTC) and siRNA1- and siRNA2-transfected U87MG cells. Fluorescence was measured per cell. (**D**) Immunofluorescence for LOXL3 (green) and nucleus (DAPI, blue) was evaluated in control (NTC) and siRNA1- and siRNA2-treated U87MG cells at 2 (D2), 4 (D4), and 7 (D7) days after transfection. Bars represent the means ± standard deviations of independent experiments. Statistically significant differences between control (NTC) and siRNA1- and siRNA2-transfected U87MG cells are represented by asterisks: **** *p* < 0.00001, *** *p* < 0.0001. Statistically significant differences between siRNA1- and siRNA2-transfected cells are represented by hashes: ### *p* < 0.0001, ## *p* < 0.001. Abbreviations: DAPI, 4′,6-diamidino-2-phenylindole; LOXL3, lysyl oxidase-like 3; NTC, nontarget control; RT-qPCR, real-time quantitative polymerase chain reaction; siRNA, small interfering RNA.

The role of LOXL3 in GBM was investigated using the U87MG cell model. As these cells express high levels of *LOXL3* (according to the HPA study) and display tumorigenic capacity, this may allow testing of selected targets using animal models in future studies. Two different siRNAs (siRNA1 and siRNA2) were used to silence *LOXL3*. Four days after transfection with these siRNAs, the cells exhibited only 15.3% and 49.1% *LOXL3* mRNA expression, respectively, versus NTC (Figure 2A). There was no difference in expression levels of LOXL3 protein analyzed by Western blotting (41.5% and 39.2% for siRNA1 and siRNA2 versus NTC, respectively) (Figure 2B). However, immunofluorescence staining of LOXL3 showed expression of 13.5% and 25.1% for siRNA1 and siRNA2, respectively (Figure 2C and 2D), relative to NTC, at day 4 after transfection. Moreover, a morphological change was observed after *LOXL3* silencing characterized by an enlargement of the tumor cell surface, which was more prominent with siRNA1 (Figure 2D). As more efficient downregulation of *LOXL3* was obtained with siRNA1, all functional analyses were conducted on day 4 after transfection with this siRNA1.

### 2.3. Transcriptome Analysis

**Figure 3:**
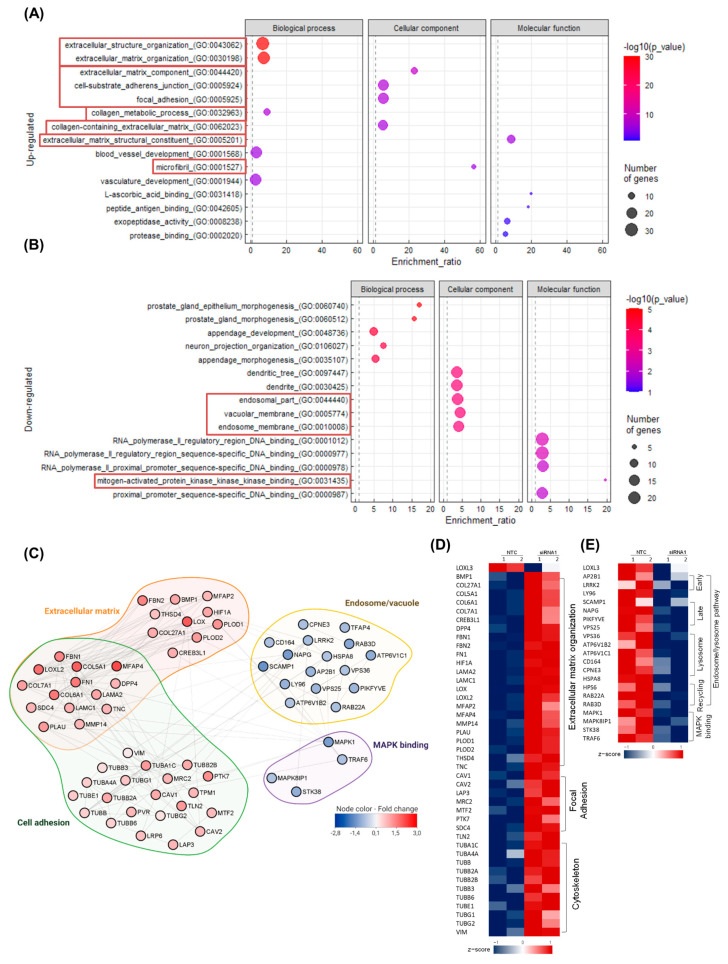
Transcriptome analysis of U87MG cell line with *LOXL3* expression knocked down by siRNA. Dot plots illustrating the top five most enriched Gene Ontology (GO) terms (biological process, cellular component, and molecular function) of differentially expressed genes (**A**) upregulated and (**B**) downregulated in siRNA1-transfected U87MG cells compared with control. Red rectangles indicate the GO processes identified in the protein–protein interaction (PPI) analysis. (**C**) The PPI network of proteins related to pathways identified in the WebGestalt analysis performed using the STRING app of the Cytoscape software. The proteins are represented by nodes, and the interactions are represented by edges (score value ≥ 0.5). Heatmaps representing the RNA-seq expression of key genes coding for proteins involved in the extracellular matrix, focal adhesion, and cytoskeleton (**D**) and endosome/lysosome and MAPK-binding proteins (**E**) in NTC and siRNA1-transfected cells. Each line represents the z-score of RPKM values. Abbreviations: LOXL3, lysyl oxidase-like 3; MAPK, mitogen-activated protein kinase; NTC, nontarget control; RNA-seq, RNA sequencing; RPKM, reads per kilobase per million; siRNA, small interfering RNA; WebGestalt, Web-Based Gene Set Analysis Toolkit.

Transcriptome analysis of *LOXL3*-siRNA1 and NTC-siRNA U87MG cells was performed to analyze the signaling pathways involved in GBM cells. In total, 15,036 genes were mapped (Table S1), and 433 differentially expressed genes (DEGs) were identified between *LOXL3*-silenced and control cells (log2 fold change ≥0.7 and ≤−0.7 and adjusted *p* ≤ 0.05 by Benjamin–Hochberg correction). Among those, 220 and 213 genes were upregulated and downregulated, respectively. The efficiency of *LOXL3* silencing was confirmed by a log2 fold change of −0.46, corresponding to 40% downregulation induced by siRNA1. The enrichment analysis of the upregulated DEGs showed a set of genes related to ECM organization (GO:0043062 and GO:0030198, both with false discovery rate (FDR) = 0 and p = 0) and collagen metabolism (GO:0032963, FDR = 4.28 × 10^−4^, *p* = 1.47 × 10^−7^) within the biological processes category. Enrichment of the ECM of DEGs was confirmed in the cellular component category, with the highest enrichment ratio (56.4) observed for microfibril (GO:0001527, FDR = 1.63 × 10^−6^, *p* = 1.25 × 10^−4^), followed by ECM (GO:0044420, FDR = 4.03 × 10^−9^, *p* = 1.37 × 10^−7^; GO:0062023, FDR = 6.37 × 10^−7^, *p* = 4.34 × 10^−5^) and adhesion processes (GO:00005924, FDR = 1.11 × 10^−7^, *p* = 4.99 × 10^−6^; GO:0005925, FDR = 1.11 × 10^−7^, *p* = 5.6 × 10^−6^). Analysis of molecular function also confirmed the differential expression of ECM genes (GO: 0005201, FDR = 1.32 × 10^−6^, *p* = 7.05 × 10^−10^) (Figure 3A). In fact, several genes coding for components of ECM microfibril, such as collagens (*COL5A1*, *COL6A1*, *COL7A1*, *COL27A1*), fibrillins (*FBN1*, *FBN2*), fibronectin (*FN1*), tenascin (*TNC*), and microfibril-associated glycoproteins (*MFAP2*, *MFAP4*), were significantly upregulated. Moreover, genes coding for enzymes, such as lysyl hydroxylases (*PLOD1*, *PLOD2*), lysyl oxidases (*LOX*, *LOXL2*), and *BMP1*, were also upregulated. Furthermore, genes coding for the intracytoplasmic focal adhesion complex, such as caveolins (*CAV1*, *CAV2*), talin 2 (*TLN2*), and calponin 2 (*CNN2*), were upregulated. This was accompanied by the upregulation of genes related to stress fibers such as CNN2 and FAP. Moreover, genes coding for caveolae plasma membrane caveolins (CAVs: *CAV1*, *CAV2*) and several tubulins, components of the cellular cytoskeleton, were upregulated (Figure 3C,D).

Downregulated DEGs were conversely enriched in cellular components corresponding to vacuoles (GO:0005774, FDR = 3.25 × 10^−4^, *p* = 1.84 × 10^−6^) and endosomes (GO:0044440, FDR = 3.25 × 10^−4^, *p* = 1.94 × 10^−6^; GO:0010008, FDR = 3.65 × 10^−4^, *p* = 2.48 × 10^−6^) (Figure 3B). The downregulated genes coded for endosomal vesicle formation, such as tumor necrosis factor receptor-associated factor 6 (*TRAF6*) and secretory carrier membrane protein 1 (*SCAMP1*); for multivesicular formation, such as components of the endosomal sorting complex required for transport II (ESCRT-II) complexes (*VPS25*, *VPS36*); and for components of vacuolar ATPases (*ATP6V1C1*, *ATP6V1B2*). Members of the RAS oncogene family involved in endosome recycling (*RAB3D*, *RAB22A*) were also downregulated. Moreover, genes coding for proteins involved in the fusion of these membrane-bound organelles, such as phosphoinositide kinase and FYVE-type zinc finger containing (*PIKFYVE*), and chaperones, such as heat shock protein family A (Hsp70) member 8 (*HSPA8*), were similarly downregulated. Additionally, the highest enrichment ratio (19.61) of downregulated genes on molecular function was associated with the mitogen-activated protein kinase (MAPK) kinase kinase binding (GO:0031435, FDR = 1.03 × 10^−2^, p = 4.39 × 10^−5^) (Figure 3B), which included four genes coding for binding proteins: mitogen-activated protein kinase 1 (*MAPK1*), serine/threonine kinase 38 (*STK38*), *TRAF6*, and mitogen-activated protein kinase 8 interacting protein 1 (*MAPK8IP1*).

We focused on the analysis of processes related to the ECM and cell adhesion for upregulated DEGs (red boxes in Figure 3A) and to endosome/vacuoles and MAPK-binding proteins for downregulated DEGs (red boxes in Figure 3B). Furthermore, the protein–protein interaction map of upregulated and downregulated DEGs was significantly connected as shown in the network constructed using the Cytoscape STRING plugin (Figure 3C). Figure 3D,E presents upregulated and downregulated DEGs connected in the protein–protein interaction network in the heatmaps.

### 2.4. LOXL3 Downregulation in U87MG Cells Decreased Viability and Invasion, Induced Death, and Increased Adhesion

**Figure 4:**
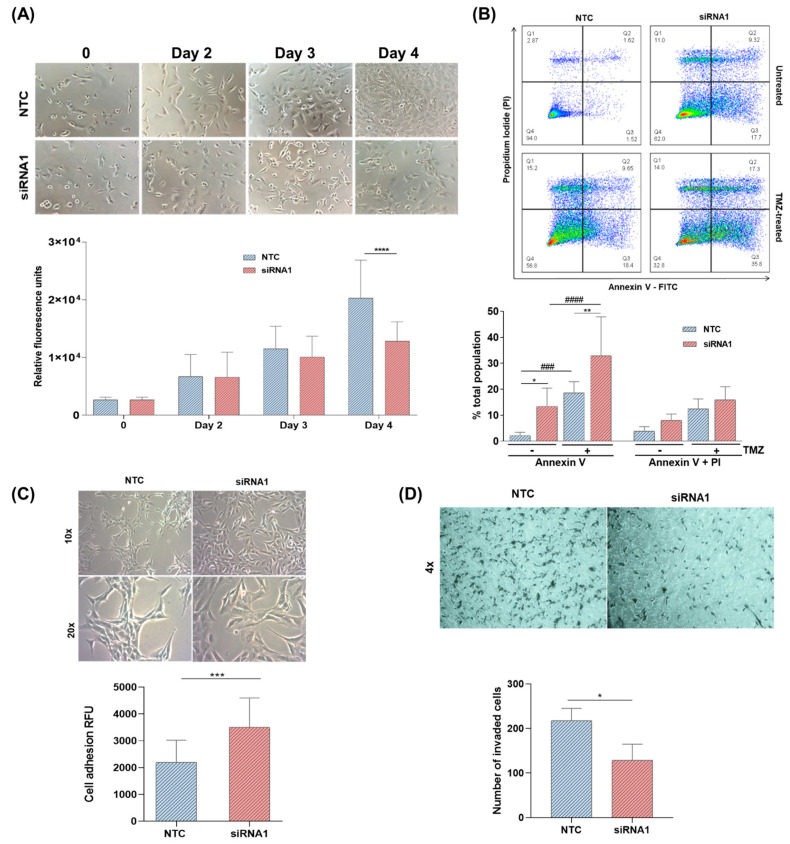
Effect of LOXL3 downregulation on cell viability and apoptosis of U87MG cells. (**A**) Representative images (magnification of 10×) and analysis of viability assay. (**B**) Flow cytometry charts and analysis of apoptosis assay using annexin V-FITC and propidium iodide staining of LOXL3-siRNA1 silenced cells compared to NTC. Apoptosis assays were performed with temozolomide (TMZ)-treated cells and compared with untreated cells. (**C**) Representative images (magnification of 10× and 20×) and analysis of cell adhesion assay of LOXL3-siRNA1 cells in comparison to control (NTC) cells. (**D**) Representative images (magnification of 4×) and analysis of cell invasion assay of LOXL3-siRNA1 cells in comparison to control (NTC) cells. Bars represent the means ± standard deviations of replicates of independent experiments. Statistically significant differences between control (NTC) and siRNA1 cells are represented by asterisks: **** *p* < 0.00001, *** *p* < 0.0001, ** *p* < 0.001, * *p* < 0.05. Statistically significant differences between cells with no treatment and cells with TMZ treatment are represented by hashes: #### *p* < 0.0001, ### *p* < 0.001.

Downregulation of *LOXL3* in U87MG cells significantly reduced cell viability compared with that observed in NTC cells at day 4 after transfection with siRNA (Figure 4A). A significant increase in annexin V-positive cells was detected by flow cytometry, indicating early cell death (Figure 4B). This response was enhanced by the costimulatory effect of TMZ, the alkylating agent used in standard care of patients with GBM. An increase of approximately 20% was observed with the combined treatment versus TMZ monotherapy (Figure 4B). Although a slight increment in late cell death, measured in cells double-stained with annexin V-FITC and propidium iodide, was observed after the downregulation of *LOXL3* and following treatment with TMZ, the differences did not reach statistical significance. Cell adhesion was also increased (Figure 4C) and cell invasion (Figure 4D) was decreased following LOXL3 downregulation versus NTC at day 4 after transfection.

### 2.5. LOXL3 Downregulation in U87MG Cells Altered Morphology and Cytoskeletal Rearrangement

**Figure 5:**
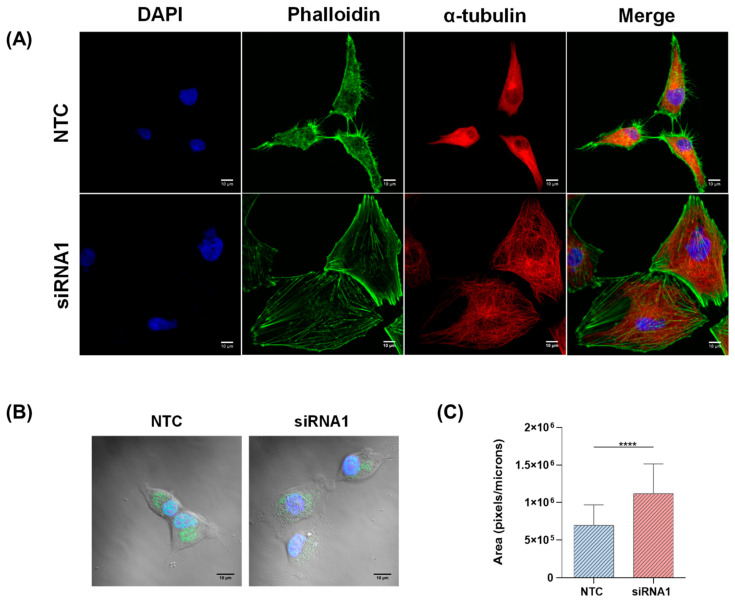
Effect of LOXL3 downregulation on cell morphology of U87MG cell line. (**A**) Representative images (magnification of 20×) of immunofluorescence staining patterns for nucleus (DAPI, blue), phalloidin (green), and α-tubulin (red) evaluated in control (NTC) and siRNA1-transfected cells after 4 days in U87MG cells. Scale bar, 10 μm. (**B**) Overlay of bright-field and fluorescence (LOXL3 and nucleus staining) representative images of control (NTC) and LOXL3-siRNA1. Scale bar, 10 μm. (**C**) Total surface area of cells presented in (**B**). Bars represent the means ± standard deviations of replicates of independent experiments. Statistically significant differences between control (NTC) and siRNA1 cells are represented by asterisks: **** *p* < 0.0001.

Additionally, when the cell cytoskeleton was visualized by actin and tubulin immunofluorescent staining 4 days after transfection with siRNA, clear morphological differences were observed between the cells with downregulated LOXL3 and NTC cells (Figure 5A). LOXL3-siRNA U87MG cells presented an increased cell surface, as demonstrated in Figure 5B, exhibiting a reorganization of the cytoskeleton with actin fibers extending to the periphery of the cells and a nonpolarized tubulin rearrangement.

### 2.6. LOXL3 Silencing in T98G Cells and Human Glioblastoma Samples

**Figure 6:**
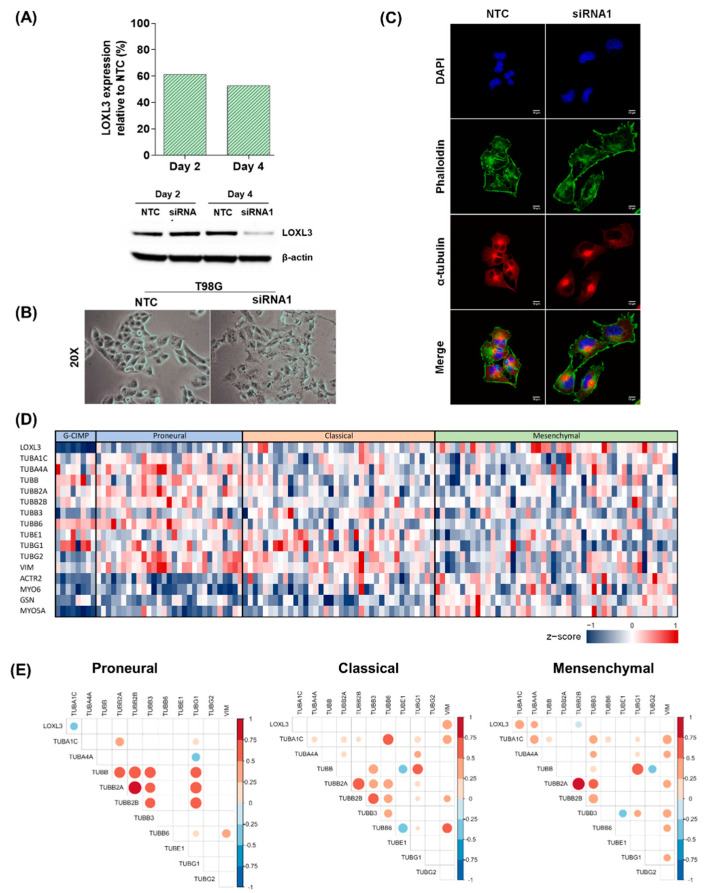
Downregulation of LOXL3 expression by siRNA in T98G cells. (**A**) LOXL3 expression relative to control (NTC) after 2 and 4 days of siRNA1 transfection analyzed by qRT-PCR and by Western blot. β-actin was used as loading control. LOXL3: 83 kDa; β-actin: 42 kDa. (**B**) Representative images (magnification of 20×) and analysis of LOXL3-siRNA1 and control (NTC) cells. (**C**) Representative images (magnification of 20×) of immunofluorescence staining patterns for phalloidin (green), α-tubulin (red), and nucleus (DAPI, blue) evaluated in control (NTC) and siRNA1-transfected cells after 4 days in T98G cells. Scale bar, 10 μm. (**D**) Heatmaps representing RNA-seq expression of GBM samples in the TCGA database for the same key genes. (**E**) Spearman correlation matrix among expression levels of genes involved in actin dynamics of GBM molecular subtypes of the TCGA database. The color bars on the right indicate the levels of correlation ranging from blue (negative correlation) to orange (positive correlation). The color intensiveness and the circle sizes are proportional to the values of r. Only the correlations with *p* < 0.05 are plotted. Abbreviations: DAPI, 4′,6-diamidino-2-phenylindole; LOXL3, lysyl oxidase-like 3; NTC, nontarget control; RT-qPCR, real-time quantitative polymerase chain reaction; siRNA, small interfering RNA; TCGA, The Cancer Genome Atlas.

T98G glioma cell line was also silenced for LOXL3 with siRNA1, with efficacy confirmed by RNA expression and protein analysis 4 days after transfection (Figure 6A). Morphological changes occurred in *LOXL3*-silenced T98G cells (Figure 6B), which exhibited enlarged cell surfaces. However, the tubulin rearrangement was not prominent compared to the observed alteration in U87MG-*LOXL3*-silenced cells (Figure 6C). Interestingly, the TCGA RNA-seq dataset of different molecular subtypes of GBM showed a significant correlation in expression between *LOXL3* and tubulin alpha (*TUBA1C* and *TUBA4A*) in the mesenchymal subtype, which was not detected in classical and proneural subtypes (Figure 6D,E).

## 3. Discussion

LOXL3, known for its function as a lysyl oxidase, has been associated with embryonic development [23,24] and diverse pathologies, including collagenopathies [25,26] and fibrosis [27]. In cancer, upregulation of LOXL3 has been detected in several tumor types such as gastric, breast, ovarian, and colorectal carcinomas and myeloproliferative tumors [14,19,20,21,22]. In fact, our in silico analyses of the TCGA transcriptome database confirmed the previously reported high expression of *LOXL3* in such tumors. These findings were also supported by a recently reported similar analysis [28]. Interestingly, GBM, the most frequent and malignant type of brain tumor in adults, was among the top 10 tumors presenting *LOXL3* overexpression. Notably, the expression levels of *LOXL3* in GBM were higher than those detected in normal brain tissue, independently of the GBM molecular subtype. Moreover, the expression levels of *LOXL3* affected the patient outcomes; higher *LOXL3* expression was associated with poorer overall survival. A similar clinical impact has been observed in patients with gastric cancer [13].

Previous studies investigating the role of LOXL3 in cancer have found an association with tumor progression and metastasis through physical interaction with SNAIL, a transcription factor involved in the epithelial–mesenchymal transition process [17] and cell proliferation [29]. Additionally, in melanoma, LOXL3 maintains genomic stability through an association with oncogenic BRAF and promotes sustained proliferation [18]. We sought to further understand the function of LOXL3 in brain tumors. Therefore, we treated the U87MG-GBM cells with *LOXL3*-siRNA to analyze the intracellular distribution and gene expression profile associated with the downregulation of *LOXL3*.

We noted a significant morphological change of U87MG cells after *LOXL3* downregulation, with enlargement of the tumor cell surface. Interestingly, the transcriptomic analysis of *LOXL3-*silenced U87MG cells showed an upregulation of genes involved in cellular focal adhesion and genes coding for cytoskeleton organization.

In fact, genes coding for the intracellular components of the focal adhesion complex and the cytoskeleton, such as *TLN2*, were upregulated with LOXL3 silencing. *TLN2* codes for a key cytoplasmic mediator of integrin adhesion to the ECM [30,31,32], while the other two genes are related to the regulation of actin filament assembly (*ENAH*) and filament organization (*CCN2*) for the modulation of cell adhesion. Furthermore, genes coding for tubulins, which polymerize into microtubules, a major component of the cell cytoskeleton, were upregulated. Microtubules act as force generators for cell protrusion, and they are involved in intracellular transport [33]. In migratory cells, the microtubule-organizing center is polarized and symmetric in front of the nucleus [34,35]. However, such characteristics were lost in the *LOXL3*-silenced U87MG cells, and a significant decrease in the invasion capacity was demonstrated in these cells. Interestingly, the TCGA RNA-seq dataset analysis demonstrated a significant correlation between the gene expression levels of *LOXL3* and two genes coding for α-tubulin *(TUBA1C*, *TUBA4A*) in the mesenchymal subtype, suggesting a prominent role of LOXL3 in the most aggressive GBM molecular subtype.

Genes related to ECM components were also upregulated following downregulation of *LOXL3*, indicating an increase in the stiffness of the cell microenvironment. Particularly, the upregulation of collagen genes (*COL5A1*, *COL6A1*, *COL7A1*, *COL27A1*), fibrillins (*FBN1*, *FBN2*), and microfibril-associated glycoproteins (*MFAP2*, *MFAP4*) pointed towards a reinforcement of the ECM microfibrillar mesh. In addition, enzymes that stabilize the cross-linking of collagen fibers, such as *PLOD1*, *PLOD2*, *BMP1*, *LOX*, and *LOXL2*, were upregulated.

Moreover, the upregulation of *FN1*, *TNC*, and plasma membrane components (e.g., *ITGAV* and *SDC4*) indicated the strengthening of the connection between the intracellular focal adhesion complex and ECM components. FN1 interacts with collagens, while ITGAV and SDC4 are associated with focal adhesion formation and FBN1 assembly, thereby contributing to microfiber deposition and subsequent elastic fiber assembly [36,37,38,39]. TNC is also involved in the interaction of FN1 and SDC4 [40]. Taken together, these results suggest that the upregulation of constituents of a highly organized elastic microfibrillar ECM provides sites for cell adhesion.

Cellular movement is a continuous and coordinated process that requires the formation and turnover of focal adhesion at the leading edge of the cell body, as well as the release of this attachment at the body and rear of the cell [41]. In fact, the focal adhesion components are continuously recycled during cell motility. Integrins are recycled by endocytosis and exocytosis through clathrin-mediated and CAV-dependent processes [42,43]. *CAV1* and *CAV2*, which code for the major components of the caveolar membrane, are upregulated DEGs. CAV1-dependent endocytosis has also been associated with FN1 turnover and negatively regulates extracellular signal-regulated kinases (i.e., ERK1 and ERK2). Interestingly, LOXL3 silencing led to the downregulation of the MAPK/ERK cascade, which is essential for focal adhesion disassembly through the FAK–paxillin complex [44,45,46]. In our LOXL3-siRNA experiment, the expression levels of paxillin were not altered. Future studies are warranted to investigate its activation through phosphorylation. Collectively, the upregulated genes after *LOXL3* silencing corroborate the observed U87MG phenotype alteration with cell spreading, increased cellular adhesion, and decreased tumor cell invasion.

Several other genes associated with the following different steps of protein recycling by endocytic trafficking and protein degradation were also downregulated in response to the silencing of *LOXL3* expression:

(1) The early step of endosomal vesicle formation (i.e., *TRAF6* and *SCAMP1*);
(2) Retrograde transport of proteins from the endosome to the trans-Golgi network (i.e., vacuolar protein sorting (*VPS25*, *VPS36*) members of the ESCRT-II complex);
(3) Endosome recycling, involving members of the RAS oncogene family (i.e., *RAB3D*, *RAB22A*);
(4) Endosome fusion to the lysosome, associated with phosphoinositide kinase (i.e., *PIKFYVE*);
(5) Maintenance of the lysosome membrane (i.e., *ATP6V1C1*, *ATP6V1B2*);
(6) Chaperone-mediated autophagy by lysosomal translocation for protein degradation (i.e., *HSPA8*).

These results may indicate that cell adhesion complex turnover through endosome vesicle trafficking, recycling, and protein degradation by the Golgi and lysosome pathways were suppressed after *LOXL3* silencing. Moreover, dysfunctional protein recycling and degradation systems may lead to the accumulation of autophagosomes and late endosomes, thereby affecting cell viability and inducing autophagy-dependent cell death [47,48].

The downregulation of the MAPK/ERK cascade may also be involved in focal adhesion disassembly through dysregulation of endosomal dynamics [49]. In fact, the expression levels of *MAPK1* (also termed ERK2), *STK38*, and *MAPK8IP1* were downregulated upon *LOXL3* silencing. These genes are related to cell growth and survival by regulation of transcription, translation, and cytoskeletal rearrangements. Upon cell detachment, STK38 is necessary for the clearance of damaged mitochondria, prevention of an increase in the levels of reactive oxygen species, and protection of cancer cells [50,51]. Moreover, STK38 regulates MYC turnover, thus extending the MYC half-life [52]. Similarly, JNK-interacting protein 1 (JIP1, also termed MAPK8IP1) is involved in JNK regulation and, consequently, in the stabilization of c-MYC protein [53,54]. Furthermore, *MAPK8IP1* is also involved in autophagosome trafficking [55], which has an impact on cell proliferation and survival. Thus, the downregulated gene expression profile of our study might be associated with the increased cell adhesion, decreased cell viability, and increased early-phase apoptosis observed in *LOXL3-*silenced U87M cells. However, further studies are necessary to confirm our hypothesis.

GBM is a very heterogeneous tumor, and in addition to the findings in U87MG, a mesenchymal subtype of GBM cell line, we also observed similar morphological change in a less aggressive GBM cell line, T98G. Further, an in-depth analysis of the role of LOXL3 in different molecular subtypes of GBM would be worthwhile to better assess its potential as a therapeutical target.

## 4. Materials and Methods

### 4.1. Public Dataset Analyses

LOXL3 mRNA expression data of different types of cancer were downloaded from the cBioPortal for Cancer Genomics (http://www.cbioportal.org, accessed on 22 July 2020) generated by RNA-seq v2 analyses of the Pan-cancer Atlas of TCGA. LOXL3 (ENSG00000115318) and its expression levels obtained by RNA-seq of cell lines from different organs were downloaded from The Human Protein Atlas (HPA) project (https://www.proteinatlas.org/, accessed on 22 July 2020). The HPA data are expressed by normalized expression values, and values ≥ 1 were considered detectable for plotting analysis. LOXL3 expression levels in GBM cases (from TCGA) and normal brain tissues (from Genotype-Tissue Expression (GTEx)), as well as the overall survival of patients with GBM, were investigated using the Gene Expression Profiling Interactive Analysis 2 (GEPIA2) online database [56].

### 4.2. Cell Culture

The glioma cell lines U87MG and T98G were obtained from the American Type Culture Collection (Manassas, VA, USA). The cells were maintained in Dulbecco’s modified Eagle’s medium (Thermo Fisher Scientific, Waltham, MA, USA), supplemented with 10% heat-inactivated fetal bovine serum (FBS) (Cultilab, Campinas, Brazil) and antibiotics (100 units/mL penicillin, 100 µg/mL streptomycin), in a humidified atmosphere with 5% CO_2_ at 37 °C. Cell line authentication was performed by short tandem repeat DNA analysis with the GenePrint 10 System (Promega, Fitchburg, WI, USA).

### 4.3. LOXL3 Silencing

Two sequences of small interfering RNA (siRNA) duplexes for LOXL3 knockdown, namely siRNA1 (5′-CGGCATGACATTGACTGTCAGTGGA-3′) and siRNA2 (5′-CTAGTTTCTGTCTCGAAGACACTGA-3′), as well as nontarget control (NTC) siRNA, were synthesized by Integrated DNA Technologies (Coralville, IA, USA). The oligonucleotides were diluted in RNase-free duplex buffer provided by Integrated DNA Technologies. U87MG and T98G cells (1 × 10^5^ cells/well) were seeded in a six-well plate and transfected with Lipofectamine RNAiMax (Thermo Fisher Scientific) after 24 h. The siRNAs for LOXL3 and NTC were used at a final concentration of 10 nM for both U87MG and T98G. LOXL3 knockdown was evaluated at 2, 4, and 7 days after transfection. The mRNA and protein levels were determined by real-time quantitative polymerase chain reaction (RT-qPCR) and Western blotting, respectively.

### 4.4. RNA Extraction and cDNA Synthesis

Extraction of RNA and DNA was performed using the RNeasy Mini Kit (Qiagen, Valencia, CA, USA) according to the protocol provided by the manufacturer. The concentration and purity of RNA were evaluated using the NanoDrop device (Thermo Fisher Scientific), and values ranging from 1.8 to 2.0 for 260:280 nm absorbance ratio denoted satisfactory purity. For cDNA synthesis, 1 µg of total RNA was required. The Maxima First Strand cDNA Synthesis Kit (Thermo Fisher Scientific) was used for the amplification, according to the instructions provided by the manufacturer. Finally, cDNA was diluted in Tris/EDTA buffer for analysis by RT-qPCR.

### 4.5. Gene Expression Analysis

The expression levels of *LOXL3* were analyzed by RT-qPCR performed on the ABI 7500 apparatus (Thermo Fisher Scientific). The primers used in this experiment were synthesized by Integrated DNA Technologies: LOXL3 (forward: CTGGAACAGGCCGCATCT; reverse: CCCCAGCATCCTCATCGT), hypoxanthine phosphoribosyltransferase (HPRT) (forward: TGAGGATTTGGAAAGGGTGT; reverse: GAGCACACAGAGGGCTACAA). These primers were designed to amplify a region containing 80–120 bp. Reactions were performed in triplicate, and the final volume was 12 µL per reaction, containing 3 µL of cDNA, 3 µL of primers (final concentration: 200 nM), and 6 µL of Power SYBR Green PCR Master Mix (Thermo Fisher Scientific). The amplification conditions included an initial incubation for 2 min at 50 °C, 10 min at 95 °C, 40 cycles of 15 s at 95 °C, and 1 min at 60 °C. The expression levels of *LOXL3* were normalized to those of the housekeeping gene HPRT. Single product amplification was confirmed by analyzing the dissociation curves. The amplification efficiencies were calculated using serial cDNA dilutions [57]. Assays were performed in duplicates and in two independent experiments. Additionally, expression levels of *LOXL3* were determined for all transfections with siRNA.

### 4.6. Western Blotting

Cell protein extracts were obtained using radioimmunoprecipitation assay (Tris-HCl 50 mM, NP-40 1%, Na-deoxycholate 0.25%, NaCl 150 mM, EDTA 1 mM) lysis buffer and a protease inhibitor cocktail (Sigma–Aldrich, St. Louis, MO, USA). Total protein concentrations were determined by the Bradford method. Cell lysates (20 µg of proteins) were separated by 4–12% gradient polyacrylamide gel electrophoresis (Thermo Fisher Scientific) in NuPAGE 3-(N-morpholino)propanesulfonic acid–sodium dodecyl sulfate electrophoresis buffer (Thermo Fisher Scientific) and transferred to a polyvinylidene difluoride membrane using the iBLOT system (Thermo Fisher Scientific). The membrane was incubated with rabbit polyclonal anti-LOXL3 (1:1000; Aviva Antibody Corporation, San Diego, CA, USA) and mouse monoclonal anti-β-actin (1:20,000; Sigma–Aldrich) as control for protein loading. Anti-rabbit and anti-mouse IgG secondary antibodies conjugated to peroxidase (1:1000; Sigma–Aldrich) and the chemiluminescence detection system Clarity Western ECL Blotting Substrate (BioRad Laboratories, Hercules, CA, USA) were used to visualize proteins in the membrane on the ImageQuant LAS4000 apparatus (GE Healthcare, Pittsburgh, PA, USA).

### 4.7. Immunofluorescence

LOXL3 localization in U87MG cells before and after transfection with siRNA was analyzed by immunofluorescence. Cells were cultured in a monolayer on poly-L-lysine-coated glass coverslips. Cells were fixed with 4% paraformaldehyde. The membrane was permeabilized with 0.1% Triton X-100, and blocking was performed with 4% goat serum. Subsequently, the membrane was incubated with the primary antibody anti-LOXL3 (1:200, Aviva Antibody Corporation) and anti-α-tubulin (1:1000, Abcam, Cambridge, UK) overnight at 37 °C, followed by incubation with the anti-rabbit IgG secondary antibody conjugated to Alexa Fluor 488 and 568 (1:400; Thermo Fisher Scientific) overnight at 4 °C. Actin filaments were stained with phalloidin conjugated to Alexa Fluor 488 (1:400; Thermo Fisher Scientific) overnight at 4 °C. Nuclei were stained with 4′,6-diamidino-2-phenylindole (DAPI; Thermo Fisher Scientific). The preparations were analyzed under Zeiss 510 LSM META and Zeiss 780-NLO confocal microscopes (Carl Zeiss Microscopy, Thornwood, NY, USA). Fluorescence quantification was performed by integrated density via the selection of regions of interest. Measurement of the total area was performed by selection in the regions of interest using bright-field microscopy. ImageJ (National Institutes of Health, Bethesda, MD, USA) software was used to perform the analyses.

### 4.8. High-Throughput Sequencing for Transcriptome Analysis

RNA-seq libraries were constructed with SureSelect Strand-Specific RNA Library Prep for Illumina Multiplexed Sequencing according to the instructions provided by the manufacturer (Agilent Technologies, Santa Clara, CA, USA). Total RNA of each sample in duplicates was used to prepare the libraries. The mean size of each library was determined on TapeStation 2200 (Agilent Technologies) with D1000 ScreenTape, and quantification was performed by RT-qPCR using Kapa Library Quantification Kit (Kapa Biosystems, Roche, Pleasanton, CA, USA). DNA libraries were pooled and sequenced on a HiSeq 2500 (Illumina, San Diego, CA, USA) with 100 bp pair-ended reads in the SELA Facility Core of School of Medicine, University of Sao Paulo. Sequencing generated an average of 51 million reads per sample. Quality control analysis was performed by FASTQC software [58]. Raw reads were aligned to the hg38 through STAR software [59]. Quantification of the gene expression data was performed using featureCounts software [60]. Data normalization was performed with edgeR software using the trimmed mean method. Expression levels were calculated using two methods: reads per kilobase per million (RPKM) and counts per million [61]. Differential expression analysis was performed using the limma framework [62]. Differentially expressed genes (DEGs; genes differentially expressed in LOXL3-knockdown U87MG cells compared with control NTC cells) were analyzed with WebGestalt (Web-Based Gene Set Analysis Toolkit), using Over-Representation Analysis and the Gene Ontology (GO) functional database [63]. RPKM data of the four samples and those of the differential expression analysis are presented in the Supplementary Material. Additionally, an enrichment map of GO terms was analyzed using the STRING plugin in Cytoscape software [64]. Functional analyses of altered genes related to processes were also performed. RPKM values were transformed to z-scores for heatmap visualization.

### 4.9. Viability Assay

A total of 1 × 10^3^ U87MG cells/well were seeded in a 96-well plate and transfected with siRNA for LOXL3 and control NTC. The cells were incubated with the PrestoBlue Cell Viability Reagent (Thermo Fisher Scientific) once daily for 4 days. Fluorescence intensity (excitation at 540 nm; emission at 560 nm) was measured using a GloMax-96 Microplate Luminometer (Promega). The background consisting of Dulbecco’s modified Eagle’s medium with 10% FBS was measured for each plate and subtracted from each measurement value. Assays were performed in octuplicate in three independent experiments.

### 4.10. Apoptosis Analysis

U87MG cells (5 × 10^3^/well) were seeded in six-well plates and transfected with siRNA for LOXL3 and NTC as previously described. On the second day post-transfection, cells were treated with 1 mM TMZ or control. Cells were labeled on the fourth day with Annexin V-FITC and propidium iodide using the Dead Cell Apoptosis kit (Thermo Fisher Scientific). A total of 30,000 events were acquired for each condition. Detection and quantification of apoptotic cells (siRNA-silenced and control) were performed by flow cytometric analysis (FACS Canto II; BD Biosciences, San Jose, CA, USA). Three independent experiments including duplicate measurements were performed.

### 4.11. Cell Adhesion and Invasion Analysis

U87MG cells (5 × 10^3^/well) were seeded in six-well plates and transfected with siRNA for LOXL3 and NTC as previously described. On the fourth day post-transfection, Dulbecco’s modified Eagle’s medium supplemented with 1% FBS was added, and cells were incubated for 2 h. Cells (5 × 10^4^/well) were seeded in 96-well plates and incubated for 3 h at 37 °C in an atmosphere containing 5% CO_2_ for the cell adhesion analysis. After three washes with phosphate-buffered saline, the cells were incubated with PrestoBlue Cell Viability Reagent (Thermo Fisher Scientific). The attached cells were quantified by measuring the fluorescence intensity at 525 nm (excitation at 560 nm) using the GloMax-96 Microplate Reader (Promega) [65,66]. Assays were performed in octuplicates in two independent experiments. Cells (1 × 10^5^/well) were seeded in transwell inserts (8 μm pore size, BD Falcon, Franklin Lakes, NJ, USA) previously prepared with Geltrex (Thermo Fisher Scientific) and rehydrated with 1% FBS for the invasion assay. Cells were seeded and incubated with 1% FBS for 20 h, using 10% FBS as chemoattractant. Invading cells were fixed with 4% paraformaldehyde and stained with 0.2% crystal violet. Invading cells were analyzed by inverted microscopy. Quantification was performed by counting all the invading cells in the inserts. Assays were performed in duplicates in two independent experiments.

### 4.12. In Silico GBM RNA-Seq Data Analyses

GBM gene expression data from the RNA-seq dataset of The Cancer Genome Atlas (TCGA) were downloaded from Genomics Data Commons Data Portal (https://portal.gdc.cancer.gov/, accessed on November 2017) and normalized by DEseq R software. Normalized read counts were converted to a z-score for heatmap visualization.

### 4.13. Statistical Analyses

For gene expression, colocalization through fluorescence intensity, cell viability, and apoptosis assays, the two-way analysis of variance test was used to compare multiple groups, followed by Tukey’s post hoc test. Student’s t-test was used to compare the groups for fluorescence quantification, cell adhesion, cell invasion, and the total surface area of cell assays. Correlation analyses between gene expression values were assessed by the nonparametric Spearman’s rho correlation test. Normality testing was performed using the Kolmogorov–Smirnov test. SPSS version 20.0 (IBM Corporation, Armonk, NY, USA) and GraphPad Prism 8 (GraphPad Software, San Diego, CA, USA) were used for statistical analysis. A *p*-value < 0.05 denoted statistical significance.

## 5. Conclusions

In summary, the DEG profile induced by LOXL3 silencing was associated with increased cell attachment, ECM stiffness, decreased cell invasion, and dysfunctional endosomal dynamics preventing cell motility. This finding suggested that dysregulation of LOXL3 interrupted the energy source needed to maintain cellular focal adhesion in sprawling tumor cells. Tumor cell invasiveness is a major characteristic of GBM. Hence, lowering LOXL3 expression may increase tumor resectability and decrease the rate of tumor recurrence, thereby improving the outcomes of patients with GBM.

## Supplementary Materials

The following are available online at https://www.mdpi.com/article/10.3390/ijms22158072/s1.
